# DIGITAL INDEPENDENCE: HEALTH-RELATED DIFFICULTY IN INTERNET USE AND HOW IT AFFECTS OLDER ADULTS’ QUALITY OF LIFE

**DOI:** 10.1093/geroni/igz038.1661

**Published:** 2019-11-08

**Authors:** Shannon Ang, Emily Lim, Rahul Malhotra

**Affiliations:** 1 University of Michigan, Michigan, United States; 2 University of Massachusetts Boston, Boston, Massachusetts, United States; 3 Duke-NUS Medical School, Singapore, Singapore, Singapore

## Abstract

Using the internet is increasingly a necessity. However, older adults may not do so due to either non-health reasons (e.g., lack of digital literacy or internet access) or health-related reasons. While researchers have studied internet use among older adults, most do not discriminate whether non-use is due to health reasons or otherwise. Recent studies also reveal that older adults use the internet to keep in touch with family and friends, highlighting that limitations in internet use may be detrimental for their well-being. We therefore, examine the key correlates of health-related difficulty in internet use, and how it may affect quality of life by reducing the size of their social support networks. Data were from a national survey of older Singaporeans (n=3966) conducted in 2016-17. Multinomial logistic regression and mediation analysis were used to identify older adult subgroups more likely to experience health-related difficulty in internet use, and whether such difficulty affected older adults’ quality of life through their social support networks. Results showed that males, those of Malay ethnicity, those with less education, and those with more instrumental activity of daily living (IADL) limitations were more likely to experience health-related difficulty in internet use. Social support networks mediated the relationship between health-related difficulty in internet use and quality of life. These findings suggest that other than managing the health conditions of older adults who face health-related difficulty in internet use, offline modes of keeping them socially connected may promote their quality of life.

